# What Do We Know About Youth Softball Pitching and Injury?

**DOI:** 10.1186/s40798-018-0168-6

**Published:** 2018-11-19

**Authors:** Jeff Barfield, Gretchen Oliver

**Affiliations:** Sports Medicine and Movement Lab, School of Kinesiology, Auburn University, 301 Wire Rd, Auburn, AL 36849 USA

**Keywords:** Fastpitch, Injury, Pitching, Softball, Windmill

## Abstract

Participation rates in fastpitch softball are continually on the rise, especially in youth. With the increased participation also comes the increased injury susceptibility. Unfortunately, the common misconception about the dynamic 360° windmill softball pitch, that it is a natural motion and thus does not cause stress on the shoulder, deterred investigation into pitching mechanics until recently. As pitching injuries in softball are on the rise, more attention is needed into the mechanics of the windmill softball pitch and injury implications. Therefore, it was the purpose of this current opinion paper to shed light on what is known about youth softball pitching and injury susceptibility.

## Keypoints

- With the increased popularity of fastpitch softball, awareness is needed on the physical demands of the windmill softball motion that relate to injury.
- Windmill softball pitchers are subject to fatigue that affects their mechanics and could potentially alter their injury susceptibility.

The windmill softball pitch is a dynamic movement that requires an athlete to rapidly move their pitching arm in a full 360° circular motion. However, a common misconception is that the windmill softball pitch produces less force on the shoulder and elbow in comparison to overhand pitching. Due to this fallacy, a lack of focus on windmill softball pitching and injury susceptibility exists among researchers, clinicians, and coaches. Since the windmill softball pitch is a dynamic motion that occurs at maximal effort due to the competitive nature of the sport, pitchers are subject to high joint loads and subsequent increased predisposition towards injury. Because of the competitive nature and increased population of the sport of softball, there has been an increased awareness regarding injury susceptibility, and anecdotally, orthopedic surgeons report an increase in diagnosed upper extremity injury for softball athletes.

Among all ages of youth, softball participation rates are on the rise. Approximately 260,000 athletes participate in Little League softball [1]. From 2014 to 2015 through 2016–2017, high school softball showed an increase from 365,528 to 368,734 athletes, respectively [2]. Additionally, participation in softball has approached that of baseball [2]. An analogous trend in injury rate exists between softball and baseball [3], with a majority of these injuries being overuse in nature. Paradoxical to the increase in participation, research regarding softball pitching mechanics and injury prevention is scarce. By raising awareness of proper mechanics, physical demands of the game, and injury propensity through attention to detail, researchers and clinicians can help create a safer playing environment for softball athletes of all ages.

## Biomechanics of the Softball Pitch

While literature covering baseball research greatly surpasses softball research, several studies have revealed that the windmill softball pitch generates similar forces about the shoulder as those experienced in overhand pitching [4, 5]. Because of these and other biomechanical similarities seen between baseball and softball, researchers and clinicians should draw on the known to help clarify the unknown about softball. Barrentine et al. [4] found that in the first half of the windmill pitch, the shoulder reached a maximum adduction and internal rotation torque as well as a maximum angular velocity of greater than 5,000°/s as the arm was approaching the top of back swing. Additionally, during the latter half of the delivery phase of the windmill softball pitch, the elbow reached a maximum compressive force of 70% of body weight as well as a maximum extension angular velocity of 570°/s. With similar joint loads found for the overhand and windmill pitching motion [4, 5], coaches and sports medicine professionals need to delve further into the investigation of why such a difference in performance demands exists between baseball and softball.

## Pitching Exposure

Recently, the performance demands of softball have been questioned in relation to injury propensity [6–9]. A major difference between the management of baseball and softball pitchers relates to the size of pitching staffs. Where a baseball pitching staff will make up half of the team with as many as 17, a softball pitching staff could include as few as one with a maximum of four [7]. In 2015, researchers concluded that the sport of softball carries a high incidence of injury, after an examination of 48 pitchers and 50 position players over the course of a season [9]. Most injuries reported within the season were not related to pitching; however, of the pitching injuries observed, more than 60% of them were to the shoulder [9]. Also considering the pitching injuries seen in that study, a majority of them occurred at the beginning of the season. Thus, the researchers concluded that softball pitching may not be as safe as it has previously been assumed. Although many factors influence an athlete’s injury susceptibility, these early season injuries observed in this study suggest more evidence of overuse of a dynamic system.

An examination of softball pitchers (14–18 years) during a 2- to 3-day tournament revealed that on average pitchers threw 1.5 games per day, 82 pitches per day for an average total of 166 pitches during the competition [8]. In this study, researchers investigated subjective measures of pain and fatigue, and they found both pain and fatigue increased from day one to day three of tournament play. Additionally, eight of nine upper extremity strength test performances decreased over the course of the weekend tournament. In another examination of fatigue and pain as well as strength and range of motion in high school softball pitchers, Yang et al. found that within 6 weeks the pitchers averaged 12 ± 5.7 games, threw an average of 89 ± 25 pitches per game, had decreased shoulder strength, and reported increased pain and fatigue [10]. In another study examining pitching a single game, researchers reported that softball pitchers 15 ± 1.2 years, threw on average 99 ± 21 pitches and experienced profound fatigue in their hip and scapula musculature following a single bout of pitching [6]. Based on these studies, clinicians, coaches, and researchers should not be surprised that softball pitchers are subject to fatigue and should set parameters around the sport of softball to help protect these athletes.

## Injuries in Pitchers

The findings of the aforementioned studies, in addition to the fact that a study of collegiate and high school fast pitch softball players from 2004 to 2009 reported approximately 370 overuse injuries [11], question the notion of why pitching restrictions have not been imposed. In a study examining rate of injuries in softball, Hill et al. [12] reported that 72.8% of the examined softball pitchers suffered injury, of which 55% involved the shoulder. In an earlier study, Loosli et al. [13] reported pitchers sustained 65% of their injuries to the upper extremity with 52.9% occurring at the shoulder. Until recently, research bypassed the relationship between kinematic data and pain history in youth and collegiate softball athletes. Softball pitching kinematic data have previously been limited to three studies [5, 14, 15]. Perhaps more research in this area will help clinicians and coaches establish pitch count guidelines for the sport of softball that are similar to those in baseball.

Analysis of collegiate pitchers over a single game found that a collegiate softball pitcher’s average pitch count was 179 ± 45 [7]. To contrast this with overhand pitching, males aged 19–22 are recommended to pitch no more than 120 pitches a day with 5 days’ rest in between outings [16]. Following a bout of this volume of softball pitching, pitchers were found to have significant decreases in hip and shoulder isometric strength but no decrease in range of motion. The decreases in isometric strength provide evidence of fatigue in the pelvic and scapular stabilizers. Given this finding, coaches and sports medicine professionals need to remember that when a muscle is fatigued, a synergist will have to take up an additional job, leading to stabilizer fatigue and overuse injury. Further investigation over a collegiate season revealed no significant differences in hip or shoulder range of motion or strength over the course of the season [7].

With the aforementioned studies reporting pain and fatigue following not only extended but acute bouts of pitching, Oliver et al. [17] examined collegiate pitchers’ pain history and pitching mechanics. The authors concluded that those with upper extremity pain have significantly different mechanics than those found to be pain free. Specifically, those with upper extremity pain displayed greater shoulder horizontal abduction at foot contact and less trunk flexion to the throwing side at ball release than the pain-free group. Furthermore, the upper extremity pain group displayed greater shoulder distraction forces at ball release. Not surprisingly, alterations in windmill pitching mechanics can alter joint kinetics which will indirectly affect injury potential for a softball pitcher.

## Conclusions

This review of literature on youth softball shows that fatigue has significant implications for softball pitchers. Sports medicine professionals know that fatigue will place athletes in compromising biomechanical positions. The fact that the windmill motion is a dynamic, competitive motion does not make it exempt. Janes and colleagues found that pitchers will use biomechanical compensations to maintain a competitive ball velocity [18]. The increased participation and injury rate for softball is clear. Clinicians and softball organizations need to answer the push for early specialization with pitch count limitations, such as those issued by Stop Sports Injuries [19], to offset the adverse effects of fatigue in youth softball pitchers.
